# A Novel Machine Learning Model for Predicting Orthodontic Treatment Duration

**DOI:** 10.3390/diagnostics13172740

**Published:** 2023-08-23

**Authors:** James Volovic, Sarkhan Badirli, Sunna Ahmad, Landon Leavitt, Taylor Mason, Surya Sruthi Bhamidipalli, George Eckert, David Albright, Hakan Turkkahraman

**Affiliations:** 1 Department of Orthodontics and Oral Facial Genetics, Indiana University School of Dentistry, Indianapolis, IN 46202, USA; jvolovic@iu.edu (J.V.); siahmad@iu.edu (S.A.); daalbri@iu.edu (D.A.); 2 Eli Lilly and Company, Indianapolis, IN 46285, USA; s.badirli@gmail.com; 3 Department of Biostatistics and Health Data Science, Indiana University School of Medicine, Indianapolis, IN 46202, USA; sbhamid@iu.edu (S.S.B.); geckert@iu.edu (G.E.)

**Keywords:** orthodontics, treatment duration, machine learning, artificial intelligence

## Abstract

In the field of orthodontics, providing patients with accurate treatment time estimates is of utmost importance. As orthodontic practices continue to evolve and embrace new advancements, incorporating machine learning (ML) methods becomes increasingly valuable in improving orthodontic diagnosis and treatment planning. This study aimed to develop a novel ML model capable of predicting the orthodontic treatment duration based on essential pre-treatment variables. Patients who completed comprehensive orthodontic treatment at the Indiana University School of Dentistry were included in this retrospective study. Fifty-seven pre-treatment variables were collected and used to train and test nine different ML models. The performance of each model was assessed using descriptive statistics, intraclass correlation coefficients, and one-way analysis of variance tests. Random Forest, Lasso, and Elastic Net were found to be the most accurate, with a mean absolute error of 7.27 months in predicting treatment duration. Extraction decision, COVID, intermaxillary relationship, lower incisor position, and additional appliances were identified as important predictors of treatment duration. Overall, this study demonstrates the potential of ML in predicting orthodontic treatment duration using pre-treatment variables.

## 1. Introduction

Orthodontic malocclusion is a common dental condition that has persisted throughout history, encompassing various forms of dental deformities, such as crowded teeth and dysfunctional occlusion [1]. The field of orthodontics comprises trained specialists who specialize in the diagnosis and treatment of individuals with dental malocclusions. Prior to commencing orthodontic treatment, a clinician must accurately diagnose the patient and devise a customized treatment plan. This plan involves the formulation of objectives aimed at attaining the highest standards of esthetics, occlusion, and long-term stability. Once these steps are completed by the orthodontist, the treatment plan is presented to the patient. In delivering the treatment plan, there is an inevitable question that comes to the patient’s mind: “How long will the braces take?” The duration of the treatment is a critical piece of information that is often most valued by the patient. Providing a credible and realistic estimate of treatment duration is of great importance, as it forms a key component of the orthodontic informed consent process and payment plan and significantly influences patient satisfaction [2,3].

Establishing an estimate of the orthodontic treatment duration begins when diagnostic records are obtained from the patient [4]. These records include dental models, photographs, and radiographs such as cephalometric and panoramic radiographs [5]. A wide range of quantitative measurements are obtained from these records, such as cephalometric values, tooth-size-arch-length discrepancy (TSALD), molar classification, etc. Seasoned orthodontists have years-worth of expertise, allowing them to plan and predict orthodontic treatment duration confidently and efficiently. However, orthodontic residents and recently certified orthodontists do not have this advantage. Although they are prepared with the technical skills to do so, it requires more time and experience.

Several studies have been developed to identify certain pre-treatment factors and their effects on orthodontic treatment duration. Aljehani et al. conducted a study that assessed the effectiveness of the American Board of Orthodontics Discrepancy Index (DI) in predicting the duration of orthodontic treatment [6]. The DI form was used to quantitatively define the complexity of an orthodontic case. They concluded that there is a weak positive correlation between DI and treatment time. Vu et al. performed a similar study that evaluated the effectiveness of the DI and the treatment complexity index (TCI), created for patients treated with fixed edgewise appliances, in predicting orthodontic treatment duration [7]. They found that the average treatment time at an orthodontic clinic was 29.10 months. It was concluded that increases in TCI or DI were significantly associated with longer treatment durations. For instance, a higher TCI score was associated with the use of headgear therapy and resulted in an average increase of 6.10 months in treatment length [7]. Finally, Mavreas et al. conducted a systematic review to investigate various factors that can affect the duration of orthodontic therapy [8]. A total of 41 articles were included in the study. They concluded that certain treatment complexities, such as extractions, impacted maxillary canines, and patient compliance, contribute to an increase in the length of orthodontic therapy. Each article reviewed in this study focused specifically on comparing a specific treatment complexity to a control. For example, Vig et al. collected data to compare the mean treatment duration of extraction versus non-extraction cases. The results showed a 5-month average increase in treatment duration in extraction cases [9]. It can be concluded from these studies that, in order to predict the duration of orthodontic therapy, accurate and complete pre-treatment data is required.

Recent advancements in the fields of artificial intelligence (AI) and machine learning (ML) offer clinicians a supplemental tool to aid in predicting orthodontic treatment duration. AI is a broad term for the technological systems that gather large data samples and export information that is used to help or improve a human’s decision-making process [10]. In recent years, there has been a remarkable surge in the application of AI and ML techniques within the field of dentistry [10,11,12], including the specialized domain of orthodontics [13,14,15,16,17,18,19,20,21,22,23,24,25]. These technologies have been harnessed to analyze radiographic images [23,26,27,28,29,30,31,32,33], predict growth [24,34,35], optimize orthodontic treatment decision-making processes [13,14,15,16,17,19,20,21,22,25,36]. Regrettably, a limited number of studies have employed AI and ML methodologies in forecasting orthodontic treatment duration [37,38]. Within this subset, Dharmasena et al. conducted a notable investigation utilizing two distinct ML algorithms, namely Naïve Bayes and Random Forest [37]. Their study focused on predicting the likelihood of either the continuation or discontinuation of orthodontic treatment, showcasing the potential of AI and ML techniques in this critical aspect of orthodontic practice. They analyzed a total of 310 records and concluded that the Random Forest algorithm had the highest accuracy in predicting continuation or discontinuation of orthodontic treatment. The variable duration of active treatment (>5 years) was concluded to be the main factor in discontinuation of treatment. 

Perhaps the most relevant study to ours examined the implementation of a ML algorithm to predict orthodontic treatment duration [38]. This study evaluated the accuracy and comparison of ML algorithms in predicting orthodontic treatment duration. It included nine different ML algorithms and eight pre-treatment variables. The study concluded that decision tree models outperformed other methods (mean square error of 54.08) and revealed that age, malocclusion, and crowding were the most influential predictors. However, this study and other existing studies on this topic have relied on a limited number of independent variables, such as DI or specific questionnaires [6,7,38]. In contrast, our study is unique in its incorporation of cephalometric data for the purpose of predicting orthodontic treatment duration and represents a novel contribution to the field. We hypothesize that ML algorithms have the capacity to predict orthodontic treatment duration in a manner comparable to that of clinicians.

## 2. Materials and Methods

### 2.1. Ethics

This study was approved as non-human subjects research (NHSR) by the Institutional Review Board (IRB) of Indiana University (Protocol #14751 14 March 2022).

### 2.2. Study Sample

The data for this retrospective study consisted of 478 patients who received orthodontic treatment at the Indiana University School of Dentistry (IUSD) Graduate Orthodontic Clinic. Inclusion criteria consisted of patients who (1) received and completed comprehensive orthodontic treatment at IUSD; (2) presented with a first molar to first molar permanent dentition; and (3) had complete pretreatment and posttreatment records. Exclusion criteria consisted of the following: (1) non-IUSD patients; (2) limited care treatments; (3) interdisciplinary cases; (4) early debonds; (5) phase I treatments; and (6) orthognathic surgery patients. The patient sample included 315 (66%) females and 163 (34%) males. 49% of the patients were treated without any extractions, while 51% were treated with extractions. There were a total of 119 (25%) patients who received treatment during the COVID pandemic. Molar classification consisted of 181 (38%) Class I, 217 (45%) Class II, and 80 (17%) Class III patients. 

### 2.3. Data Collection

**Figure 1:**
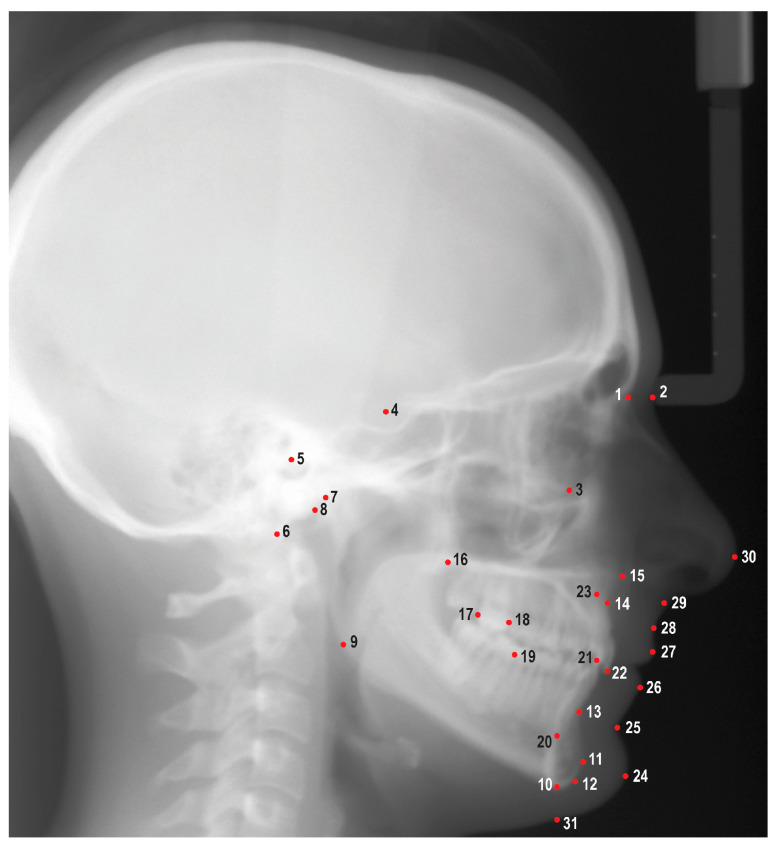
Cephalometric landmarks used in this study. 1: Nasion (N), 2: Soft tissue nasion (N’), 3: Orbitale (Or), 4: Sella (S), 5: Porion (Po), 6: Basion (Ba), 7: Condylion (Co), 8: Articulare (Ar), 9: Gonion (Go), 10: Menton (Me), 11: Pogonion (Pog), 12: Gnathion (Gn), 13: B point (B), 14: A point (A), 15: Anterior nasal spine (ANS), 16: Posterior nasal spine (PNS), 17: Distal of upper first molar (U6d), 18: Mesial of upper first molar (U6m), 19: Mesial of lower first molar (L6m), 20: Lower incisor root apex (L1a), 21: Lower incisor incisal edge (L1i), 22: Upper incisor incisal edge (U1i), 23: Upper incisor root apex (U1a), 24: Soft tissue pogonion (Pog’), 25: Soft tissue B point (B’), 26: Lower lip (Li), 27: Upper lip (Ls), 28: Soft tissue A point (A’), 29: Subnasale (Sn), 30: Pronasale (Pn), 31: Soft tissue menton (Me’).

An experienced orthodontic faculty member (HT) and three orthodontic residents (JV, TM, LL) attended three calibration sessions prior to reviewing and tracing the cephalometric radiographs. A total of 31 cephalometric landmarks were identified using Dolphin Imaging Software (Patterson Dental, Saint Paul, MN, USA) (Figure 1). These cephalometric landmarks were used to generate a cephalometric analysis that included 46 linear and angular measurements (Table 1). Demographic and treatment information, including age, gender, race, ethnicity, actual treatment time, additional appliances, and COVID factor, was gathered from the IUSD electronic practice management software (Axium v.7.09.00.45, Exan Software, Las Vegas, NV, USA). Additional appliance factors included impacted canines, expanders, and headgear. COVID factor was included if the patient was being treated during the pandemic when IUSD limited elective dental appointments (March 2020–June 2020). Maxillary/mandibular TSALD and molar classifications were collected by utilizing pretreatment photographs and digital casts. TSALD was categorized into no crowding/spacing (<1 mm), mild crowding/spacing (1–3 mm), moderate crowding/spacing (4–7 mm), and severe crowding/spacing (>8 mm). 

### 2.4. Reliability Assessment

In order to evaluate the agreement among examiners, as well as the repeatability within examiners, a total of twenty patient records were randomly selected for each resident. These records were subsequently retraced to assess intra-examiner repeatability and inter-examiner agreement by using the intraclass correlation coefficient (ICC).

### 2.5. Training and Testing the Models

Following the data collection, the statistician randomly distributed the patient sample into a training set, which comprised two-thirds of the total sample, and a test set, which constituted one-third of the total sample. The aforementioned training and test sets were used to both train and test each of the designated ML algorithms. 

A total of 8 traditional regression models and a small multilayer perceptron (MLP), namely a neural network, were used to predict the orthodontic treatment duration. The implemented models include 4 linear models (Linear Regression, Lasso, Ridge, and Elastic Net), 2 tree-based models (XGBoost and Random Forest), 2 kernel-based models (Support Vector Regression (SVR) and Gaussian Process Regression), and a neural network (MLP Regressor). Since the dataset contains both numeric and categorical values with different feature scales, tree-based methods are a natural choice. To explore both linear and non-linear relationships between covariate and treatment months, we extended ML methods to linear and kernel-based methods. For the sake of completeness, we added a small neural network to the pack, although the size of the dataset is quite small for this data hungry approach. Finally, we performed automated hyperparameter tuning using the Python Hyperopt package for each model. 

Due to the number of numerical features in this study, it was determined to test and train the ML methods utilizing both the raw data and the normalized data set. Normalization is an important step when training traditional ML methods. It is particularly important for kernel-based methods like SVR, as they are sensitive to outliers, and normalization mitigates the effect of outliers. Linear models also benefit from normalization, and model interpretability becomes easier as the features now reside on a common scale. Normalization makes sure the variance and scale of some features do not overshadow the relative importance of other features. For these reasons, we determined to employ min-max normalization on the data and the raw data separately.

### 2.6. Statistical Analysis

Descriptive statistics were provided for normalized and raw data groups for both true and absolute differences. For both normalized and scaling analyses, means with 95% confidence intervals were provided for differences between the actual and initial measurements, and each initial measurement was provided for both true and absolute differences. Mean absolute error (MAE), root mean square error (RMSE), and mean error (ME) were calculated to further evaluate the accuracy of the ML algorithms. A one-sample *t*-test was used to test for the difference from zero. One-way analysis of variance (ANOVA) with a random effect was used to test for the differences between the 9 methods for both normalized and raw data groups. For both groups, intraclass correlation coefficients (ICCs) and Bland–Altman plots were used to measure the agreement between actual and final measurements. All the tests were conducted at a 5% significance level. All the analyses were done using SAS 9.4 software (SAS Institute Inc., Cary, NC, USA).

## 3. Results

### 3.1. Reliability Analysis

The reliability analysis conducted in this study assessed the repeatability and agreement of 50 measurable pre-diagnostic variables, and the results are given in Table 2. The findings indicate that 80% of these variables demonstrated excellent (ICCs > 0.90) or good (0.75 < ICCs < 0.90) intra-examiner repeatability, highlighting the consistent and reliable nature of the measurements performed by the same examiner [39]. Furthermore, inter-examiner agreement was evaluated, with 86% of the variables showing excellent or good agreement between different examiners. These results provide evidence of the robustness and consistency of the measurements, supporting the reliability of the data used in the study.

### 3.2. Descriptive Statistics

The study encompassed a sample population with a mean age of 16.00 ± 9.32 years. The average duration of treatment was found to be 30.12 ± 9.32 months. Moreover, the mean ANB value for the sample was determined to be 3.29° ± 2.06, while the average SN-MP measurement stood at 32.66° ± 5.98. Furthermore, a comparative analysis revealed that the average treatment time for cases involving extraction was 33.46 ± 8.94 months, whereas non-extraction cases exhibited an average treatment time of 26.58 ± 8.38 months. Table 3 provides the complete results for the descriptive statistics, including the mean, standard deviation, and minimum and maximum values.

### 3.3. Performance of ML Models

The performance of the ML models, including MAE, RMSE, ME, and ICCs, is presented in Table 4. Bland–Altman plots showing the agreement between actual and predicted treatment durations using raw and normalized data are presented in Figures S1 and S2. MAE was selected as the preferred accuracy metric, as it provides a reliable measure of the ML performance in predicting orthodontic treatment duration. The results highlight the consistent performance of the linear models in our study. This observation may be attributed to two factors: either the available data does not contain enough information to capture non-linear relationships, or the data itself inherently follows a linear trend. Also, the impact of data normalization on gaussian regression is evident, as it significantly influenced the results.

**Figure 2:**
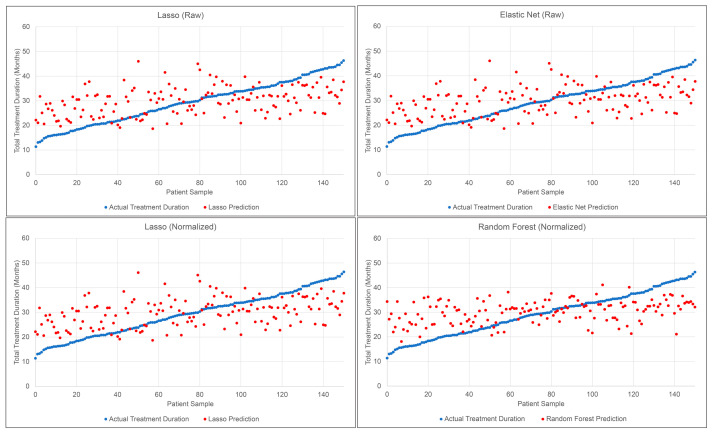
Actual treatment time vs. ML predictions for the test set.

**Figure 3:**
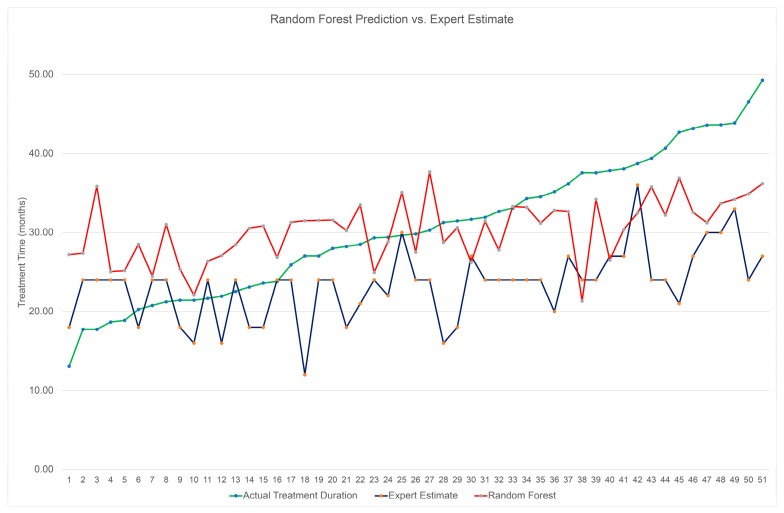
Comparison of expert estimates, RF predictions, and actual treatment duration.

Figure 2 provides the actual vs. predicted treatment times for the two most accurate ML methods for the raw data set (Lasso and Elastic Net) and the two most accurate ML models for the normalized data set (Lasso and Random Forest). The graphs reveal that the ML methods appear to overestimate the prediction for the shorter actual treatment times and underestimate the prediction for the longer treatment times. Figure 3 represents 51 samples of the Random Forest test set compared to the expert estimate provided to the patient prior to treatment. The expert estimate was determined by IUSD orthodontic residents and faculty members. The actual treatment time was organized chronologically, which is the reason for the upward trend.

### 3.4. Predictive Features

**Figure 4:**
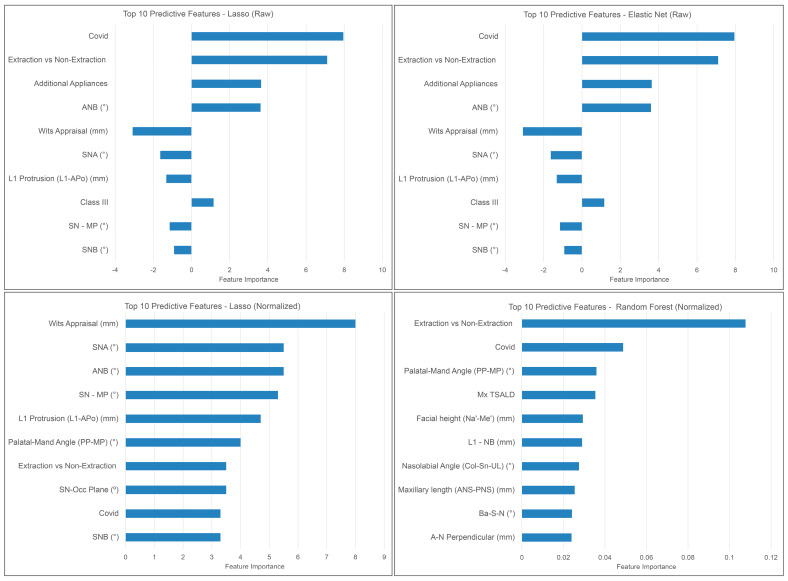
Most predictive features picked up by the top-performing ML methods.

Figure 4 shows the most predictive features picked up by the top-performing ML models: Elastic Net, Random Forest, and Lasso. The extraction decision, the impact of COVID-19, and the utilization of additional appliances consistently emerged as the most influential features in predicting orthodontic treatment duration. These features exhibited notable consistency in their appearance across multiple ML models, reinforcing their significance in accurately estimating treatment duration.

### 3.5. Method Comparison 

Table 5 represents the ANOVA comparing the performance of the tested models in predicting the actual orthodontic treatment duration. A significant difference exists between the Gaussian process, SVR, and MLP regressor when compared to the remaining ML algorithms. This disparity suggests notable variations in their predictive capabilities or performance outcomes.

## 4. Discussion 

The study aimed to assess and analyze the feasibility of developing a reasonably accurate ML model for predicting orthodontic treatment duration based on pretreatment diagnostic variables. Estimating treatment duration in orthodontics requires extensive expertise and discipline-specific knowledge. The development of such a model holds significant potential for optimizing treatment planning and facilitating effective communication between orthodontists and patients [2]. By offering a ML model that can provide reliable predictions, informed consent for patients can be enhanced, leading to increased levels of patient satisfaction. In addition to providing precise treatment estimates, it is equally essential to establish an accurate range. 

The present study revealed a mean treatment duration of 30.12 ± 9.32 months. Notably, Vu et al. reported comparable treatment durations of 29.10 ± 10.99 months within an orthodontic department [7]. Specifically, extraction cases exhibited an average treatment time of 33.15 months, whereas non-extraction cases had an average treatment time of 26.90 months. Similar trends were observed in the investigation conducted by Holman et al., wherein extraction cases had an extended average treatment time of 30 months, while non-extraction cases exhibited a shorter average treatment duration of 26 months [40]. Moreover, patients undergoing orthodontic treatment during the COVID pandemic experienced an average treatment time increase of 3 months. This finding aligns with the study conducted by Morosan, which reported a comparable treatment delay of 2 months [41].

A comprehensive evaluation was conducted in this study to assess the performance of different ML models in predicting orthodontic treatment duration. ML models were able to predict the actual orthodontic treatment duration within ±7.27 months. A consistent pattern was observed among the ML models, with a tendency to overestimate shorter treatment times while underestimating longer treatment times. This behavior may stem from the algorithms’ inclination to converge treatment time predictions towards the average total treatment duration of the patient sample. In order to evaluate the clinical performance of the ML models, it is essential to establish a benchmark against which their predictive accuracy can be evaluated. The benchmark was established from a subset of 148 patients that had received expert-estimated treatment time prior to commencing orthodontic treatment. The analysis of expert predictions against the actual treatment time revealed a MAE of ±9.66 months. Notably, the expert predictions demonstrated a 25% decrease in accuracy compared to the best ML models. This emphasizes the fact that predicting orthodontic treatment duration is a challenging task for both ML models and clinicians. 

The ML models employed in this study were evaluated to identify the most influential features for treatment duration prediction. The analysis of the best-performing ML models revealed that extraction decision, COVID-19 impact, additional appliances, intermaxillary relationship, lower incisor position, and vertical skeletal pattern were the features accorded the highest weights in treatment time prediction. This observation aligns with the findings of Fisher et al., who also identified extraction decision, lower incisor position, and vertical skeletal pattern as significant factors impacting orthodontic treatment duration [42]. Similarly, Mavreas et al. reported an extended treatment duration associated with additional appliances and extraction treatments. As for the COVID-19 impact, the temporary closure of orthodontic clinics for approximately three months undoubtedly had a substantial impact on treatment duration, as patients were unable to continue their regular appointments and adjustments during this period. Interestingly, our study and that of Mavreas both indicated that molar classification and age did not exert significant effects on treatment time [8]. The inclusion criteria encompassed all patients with permanent dentition, potentially diminishing the influence of age when the ML models assigned importance to specific features. Moreover, the majority of patients in our sample shared a similar age range. Regarding molar classification, its impact may have been limited due to the absence of malocclusion severity among the pre-treatment variables. For example, patients classified as a quarter-step Class II received the same weight as those classified as a full-step Class II.

Our findings revealed notable variations in the performance of the methods, with Elastic Net, Lasso, and Random Forest models emerging as the top performers. Conversely, the MLP regressor, Gaussian process, and SVR models exhibited comparatively poorer predictive capabilities. Notably, linear regression and tree-based models exhibited superior performance, while kernel-based and deep learning models yielded slightly less accurate predictions. Several factors, including sample size, feature selection, and regularization techniques, likely contributed to these disparities. In the context of limited sample sizes, the simplicity and reduced risk of overfitting in linear models make them more likely to outperform non-linear models. This advantage stems from the stable and reliable estimations of variable relationships that linear models can provide. Elnagar et al. yielded comparable results, highlighting advanced decision tree regression models as the most effective among ML models [38]. Their study also quantified accuracy through mean square error, yielding a value of 54.08. Notably, unlike our research, Elnagar et al.’s investigation did not encompass cephalometric measurements. However, their five most important features were identified as patient age, upper/lower crowding, overjet, and AI score (treatment difficulty estimated by AI) [38]. 

It is essential to recognize that orthodontic treatment duration is influenced by various factors, encompassing both pre-diagnostic measurable data and factors that arise during the course of treatment. The pre-diagnostic data, obtained through clinical examinations and cephalometric analyses, contribute to the complexity of each case, ultimately impacting the duration of treatment. This study primarily focuses on analyzing these pre-diagnostic variables. However, it is crucial to acknowledge that additional factors that arise during treatment, such as patient compliance, treatment-related emergencies, and missed appointments, can significantly influence the overall treatment duration. Beckwith’s study found that factors such as broken brackets, poor oral hygiene, and missed appointments had a significant increase in orthodontic treatment duration [43]. Furthermore, the MAE values obtained in this study may provide insight into the extent to which these treatment-related factors can affect the treatment duration. By examining the MAE values, we can gain a better understanding of the interplay between pre-diagnostic factors (cephalometric analysis, crowding, molar classification, etc.) and the additional factors that emerge during treatment (compliance, missed appointments, etc.), contributing to a comprehensive assessment of orthodontic treatment duration.

This study is limited by its inability to account for these factors. Furthermore, the sample size represents another limitation, as ML methods tend to exhibit improved performance when provided with larger datasets. Future investigations should prioritize enlarging the sample size, which should include a wider range of demographics, and exploring the potential benefits of incorporating image detection of cephalometric radiographs, which could mitigate concerns regarding intra-examiner reliability and inter-examiner agreement.

## 5. Conclusions

All tested ML models were able to predict orthodontic treatment duration within a clinically acceptable range. Although ML models had similar accuracy, linear models and Random Forest were the most predictive models, while SVR and Gaussian process regression were the least. The extraction decision, COVID factor, intermaxillary relationship, lower incisor position, and additional appliances were found to be the most predictive features in determining treatment time.

## Figures and Tables

**Table 1:** Cephalometric variables and their definitions.

Category	Measurements	Definitions
Maxilla to Cranial Base	SNA (°)	The angle formed by connecting the sella, nasion, and A point.
	SN-Palatal Plane (°)	The angle formed from the intersection of the sella-nasion line and a line drawn connecting the anterior nasal spine to the posterior nasal spine.
	SN-Occlusal Plane (°)	The angle formed from the sella-nasion and occlusal plane.
	A-N Perpendicular (mm)	The linear distance from A point to the nasion perpendicular.
Mandible to Cranial Base	SNB (°)	The angle formed by connecting the sella, nasion, and B point.
	SNPg (°)	The angle formed by connecting the sella, naison, and pogonion.
	FMA: MP-FH (°)	The angle formed from the intersection of the porion-orbitale line and a line drawn connecting the gonion to the gnathion.
	SN-MP (°)	The angle formed from the intersection of the sella-nasion line and a line drawn connecting the gonion to the gnathion.
	Mandibular Plane to Occlusal Plane (°)	The angle formed by the mandibular plane and the occlusal plane.
	B-N Perpendicular (mm)	The linear distance from the B point to the nasion perpendicular.
	Pog-N Perpendicular (mm)	The linear distance from the pogonion to the nasion perpendicular.
	Y-Axis: SGn-SN (°)	The angle formed by connecting the nasion, sella, and gnathion.
Maxilla to Mandible	ANB (°)	The difference between SNA and SNB.
	Palatal-Mandibular Angle (PP-MP) (°)	The angle formed from the palatal plane and the mandibular plane.
	Wits Appraisal (mm)	The distance between A point to the occlusal plane and B point to the occlusal plane.
	Maxillary Length: ANS-PNS (mm)	The linear measurement between the anterior nasal spine and the posterior nasal spine.
	Mandibular Length: Co-Gn (mm)	The linear measurement between the condylion and the gnathion.
Cranial Base	Cranial Base Flexure Angle: Ba-S-N (°)	The angle formed by connecting the basion, sella, and nasion.
Upper Incisors to Maxilla	U1-SN (°)	The angle formed by a line connecting the sella and the nasion and a line connecting the upper incisor incisal tip to the root apex.
	U1-NA (°)	The angle formed by a line connecting the nasion to the A point and a line connecting the upper incisor incisal tip to the root apex.
	U1-NA (mm)	The linear measurement from the labial surface of the upper incisor to the line connecting the nasion to the A point.
	U1-Palatal Plane (°)	The angle formed by the position of the maxillary incisor to the palatal plane.
	U1 Protrusion (U1-APo) (mm)	The distance from the maxillary incisor to the A point-pogonion reference line.
Lower Incisors to Mandible	L1-MP (°)	The angle formed by a line connecting the lower incisor incisal tip to the root apex and a line connecting the gonion to the gnathion.
	L1-NB (°)	The angle formed by a line connecting the lower incisor incisal tip to the root apex and a line connecting the nasion to the B point.
	L1-NB (mm)	The linear measurement from the labial surface of the lower incisor incisal to the line connecting the nasion to the B point.
	L1 Protrusion (L1-Apo) (mm)	The distance from the mandibular incisor to the A point-pogonion reference line.
Incisors to Each Other	Interincisal Angle (°)	The angle formed by a line connecting the lower incisor incisal tip to the apex and a line connecting the upper incisor incisal tip to the root apex.
	Overjet (mm)	The horizontal distance from maxillary incisor tip to mandibular incisor tip.
	Overbite (mm)	The vertical distance from the maxillary incisor tip to the mandibular incisor tip.
Soft Tissue	Upper Lip to E-Plane (mm)	The linear distance from the upper lip to a line connecting the soft tissue pogonion and pronasale.
	Lower Lip to E-Plane (mm)	The linear distance from the lower lip to a line connecting the soft tissue pogonion and pronasale.
	ILG (HP) (mm)	The vertical distance from stomion superius to stomion inferius.
	Nasolabial Angle (Pn-Sn-UL) (°)	The angle formed by the pronasale, subnasale, and upper lip.
	H-Angle (Pg’UL-Pg’Na’) (°)	The angle formed by soft tissue pogonion-upper lip to soft tissue pogonion-soft tissue nasion.
	Facial Height (Na’-Me’) (mm)	The linear measurement from soft tissue nasion and soft tissue menton.
	Soft Tissue Upper Face Height: G’-Sn’ (mm)	The linear measurement between soft tissue glabella and soft tissue subnasale.
	Soft Tissue Lower Face Height: Sn’-Me’ (mm)	The linear measurement between soft tissue subnasale and soft tissue pogonion.
	Hard Tissue Upper Face Height: N-ANS (mm)	The linear measurement between the nasion and anterior nasal spine.
	Hard Tissue Lower Face Height: ANS-Me (mm)	The linear measurement between the anterior nasal spine and menton.
	UFH (N-ANS/(N-ANS + ANS-Me)) (%)	The ratio of the upper face height to facial height.
	LFH (ANS-Me/(N-ANS + ANS-Me)) (%)	The ratio of lower face height to facial height.
	Posterior Face Height: Ar-Go (mm)	The linear measurement between articulare and gonion.
	PFH:AFH (Co-Go:N-Me) (%)	The ratio of posterior facial height to anterior facial height.
Profile	Convexity: NA-APo (°)	The angle formed by connecting the nasion, A point, and pogonion.
	Facial Angle: FH-NPo (°)	The angle formed by a line connecting the porion to the orbitale and a line connecting the nasion to the pogonion.

**Table 2:** Results of the reliability analyses.

	Threshold	Agreement	Quantity	Percent
	0 < ICCs < 0.50	Poor	3	6%
Intra-examiner repeatability	0.50 < ICCs < 0.75	Moderate	7	14%
	0.75 < ICCs < 0.90	Good	14	28%
	ICCs > 0.9	Excellent	26	52%
	0 < ICCs < 0.50	Poor	1	2%
Inter-examiner agreement	0.50 < ICCs < 0.75	Moderate	6	12%
	0.75 < ICCs < 0.90	Good	27	54%
	ICCs > 0.9	Excellent	16	32%

**Table 3:** Descriptive statistics for the pre-treatment variables.

Variable	Mean	SD	Min	Max
Treatment Time (Months)	30.12	9.32	11.37	51.80
Age (Years)	16.00	5.61	9.00	50.00
SNA (°)	82.55	4.14	71.00	94.90
SN-Palatal Plane (°)	7.52	3.71	−2.80	19.20
SN-Occlusal Plane (°)	15.62	4.85	−1.50	29.00
A-N Perpendicular (mm)	0.49	3.72	−9.60	12.00
SNB (°)	79.25	4.14	67.40	92.90
SNPg (°)	79.78	4.18	66.50	93.00
FMA (MP-FH) (°)	27.28	5.54	11.00	45.50
SN—MP (°)	32.66	5.98	14.80	51.70
Mandibular Plane to Occlusal Plane (°)	18.25	4.52	4.80	35.10
B-N Perpendicular (mm)	−4.36	6.09	−22.10	13.20
Pog-N Perpendicular (mm)	−4.09	7.01	−24.80	15.70
Y-Axis (SGn-SN) (°)	67.84	4.27	56.20	83.90
ANB (°)	3.29	2.06	−3.70	11.10
Palatal-Mandibular Plane Angle (PP-MP) (°)	26.35	6.08	2.10	43.10
Wits Appraisal (mm)	−0.15	3.06	−9.50	8.90
Maxillary length (ANS-PNS) (mm)	49.47	3.85	38.20	65.40
Mandibular length (Co-Gn) (mm)	113.10	8.20	93.40	158.40
Ba-S-N (°)	130.91	5.71	114.30	153.90
U1—SN (°)	107.44	9.60	67.30	134.50
U1—NA (°)	24.89	8.80	−14.10	51.50
U1—NA (mm)	5.55	3.22	−7.50	17.70
U1—Palatal Plane (°)	114.96	8.82	80.40	140.50
U1 Protrusion (U1-APo) (mm)	7.74	3.74	−2.40	20.90
L1—MP (°)	92.19	7.70	65.50	112.80
L1—NB (°)	26.70	8.35	4.80	51.30
L1—NB (mm)	5.60	3.21	−1.30	17.10
L1 Protrusion (L1-APo) (mm)	3.35	3.41	−5.20	13.60
Interincisal Angle (U1-L1) (°)	125.12	14.53	89.50	171.60
Upper Lip to E-Plane (mm)	−1.60	3.12	−12.90	8.50
Lower Lip to E-Plane (mm)	0.37	3.64	−9.80	13.80
Interlabial gap (HP) (mm)	1.19	1.40	−1.10	8.80
Nasolabial Angle (Col-Sn-UL) (°)	106.58	11.27	68.00	132.60
Holdaway Angle (Pg’UL-Pg’Na’) (°)	17.34	4.91	0.60	36.70
Facial height (Na’-Me’) (mm)	112.97	7.60	79.90	137.20
Soft tissue Upper Facial Height (G’-Sn’) (mm)	63.96	4.82	49.60	76.80
Soft tissue Lower Facial Height (Sn’-Me’) (mm)	69.64	6.18	53.60	87.10
Upper Face Height (N-ANS) (mm)	49.13	3.41	38.50	59.60
Lower Face Height (ANS-Me) (mm)	63.37	6.34	44.90	94.20
UFH (N-ANS/(N-ANS + ANS-Me)) (%)	43.75	2.56	36.40	50.10
LFH (ANS-Me/(N-ANS + ANS-Me)) (%)	56.25	2.56	49.90	63.60
Posterior Face Height (Ar-Go) (mm)	42.89	5.24	29.70	62.70
PFH:AFH (Co-Go: N-Me) (%)	51.74	4.84	37.40	67.30
Convexity (NA-APo) (°)	5.65	5.30	−8.20	26.30
Facial Angle (FH-NPo) (°)	87.76	3.83	75.30	98.20
Overjet (mm)	4.48	2.33	−2.30	18.20
Overbite (mm)	2.17	2.19	−6.30	10.20

SD: Standard deviation; Min: minimum; Max: maximum.

**Table 4:** Results for the ML predictions and performance assessments.

	Raw Data				Normalized Data			
	MAE	RMSE	ME	ICC	MAE	RMSE	ME	ICC
XGBoost	8.70	10.56	−0.29	0.97	8.43	10.40	−0.45	0.97
Random Forest	7.75	9.63	−0.65	0.97	7.27	8.79	−0.46	0.96
Lasso	7.27	8.73	−0.13	0.96	7.27	8.73	−0.13	0.96
Ridge	7.30	8.76	−0.29	0.96	7.29	8.73	−0.16	0.96
Linear Regression	7.30	8.76	−0.29	0.96	7.31	8.77	−0.36	0.96
Elastic Net	7.27	8.73	−0.13	0.96	7.27	8.73	−0.13	0.96
Gaussian Process	29.77	31.27	29.77	0.99	8.62	11.02	3.42	0.98
Support Vector	7.66	9.13	1.18	0.96	10.24	12.47	0.07	0.98
MLP Regressor	13.04	15.69	12.63	0.99	8.52	10.57	−3.20	0.97

MAE: Mean absolute error; RMSE: Root mean square error; ME: Mean error; ICC: Intra-class correlation coefficient.

**Table 5:** ANOVA comparison for the ML models’ performance.

Methods	Estimate	Standard Error	*p*-Value
Elastic Net < Gaussian	−1.35	0.47	<0.01
Elastic Net and Lasso	0.00	0.47	1.00
Elastic Net and Linear	−0.03	0.47	0.94
Elastic Net < MLP	−1.25	0.47	0.01
Elastic Net and Random Forest	0.00	0.47	1.00
Elastic Net and Ridge	−0.01	0.47	0.97
Elastic Net < SVR	−2.96	0.47	<0.01
Elastic Net < XGBoost	−1.16	0.47	0.01
Gaussian > Lasso	1.35	0.47	<0.01
Gaussian > Linear	1.31	0.47	0.01
Gaussian and MLP	0.10	0.47	0.83
Gaussian > Random Forest	1.35	0.47	<0.01
Gaussian > Ridge	1.33	0.47	<0.01
Gaussian < SVR	−1.62	0.47	<0.01
Gaussian and XGBoost	0.19	0.47	0.68
Lasso and Linear	−0.03	0.47	0.94
Lasso < MLP	−1.25	0.47	0.01
Lasso and Random Forest	0.00	0.47	1.00
Lasso and Ridge	−0.01	0.47	0.97
Lasso < SVR	−2.96	0.47	<0.01
Lasso < XGBoost	−1.16	0.47	0.01
Linear < MLP	−1.21	0.47	0.01
Linear and Random Forest	0.03	0.47	0.94
Linear and Ridge	0.02	0.47	0.97
Linear < SVR	−2.93	0.47	<0.01
Linear < XGBoost	−1.12	0.47	0.02
MLP > Random Forest	1.25	0.47	0.01
MLP > Ridge	1.23	0.47	0.01
MLP < SVR	−1.72	0.47	<0.01
MLP and XGBoost	0.09	0.47	0.85
Random Forest and Ridge	−0.01	0.47	0.97
Random Forest < SVR	−2.96	0.47	<0.01
Random Forest < XGBoost	−1.16	0.47	0.01
Ridge < SVR	−2.95	0.47	<0.01
Ridge < XGBoost	−1.14	0.47	0.01
SVR > XGBoost	1.81	0.47	<0.01

## Data Availability

Data was gathered from the Indiana University School of Dentistry, Department of Orthodontics.

## Supplementary Materials

The following supporting information can be downloaded at: https://www.mdpi.com/article/10.3390/diagnostics13172740/s1, Figure S1: Bland–Altman plots showing the agreement between actual and predicted treatment durations using raw data; Figure S2: Bland–Altman plots showing the agreement between actual and predicted treatment durations using normalized data.
