# Supplementary material for: A Novel Machine Learning Model for Predicting Orthodontic Treatment Duration
Source: Diagnostics (Basel). 2023 Aug 23;13(17):2740. doi: 10.3390/diagnostics13172740 (PMC10486486; doi:10.3390/diagnostics13172740)
Supplement: Supplementary file 1 [file diagnostics-13-02740-s001.zip › diagnostics-2503638-supplementary.pdf]

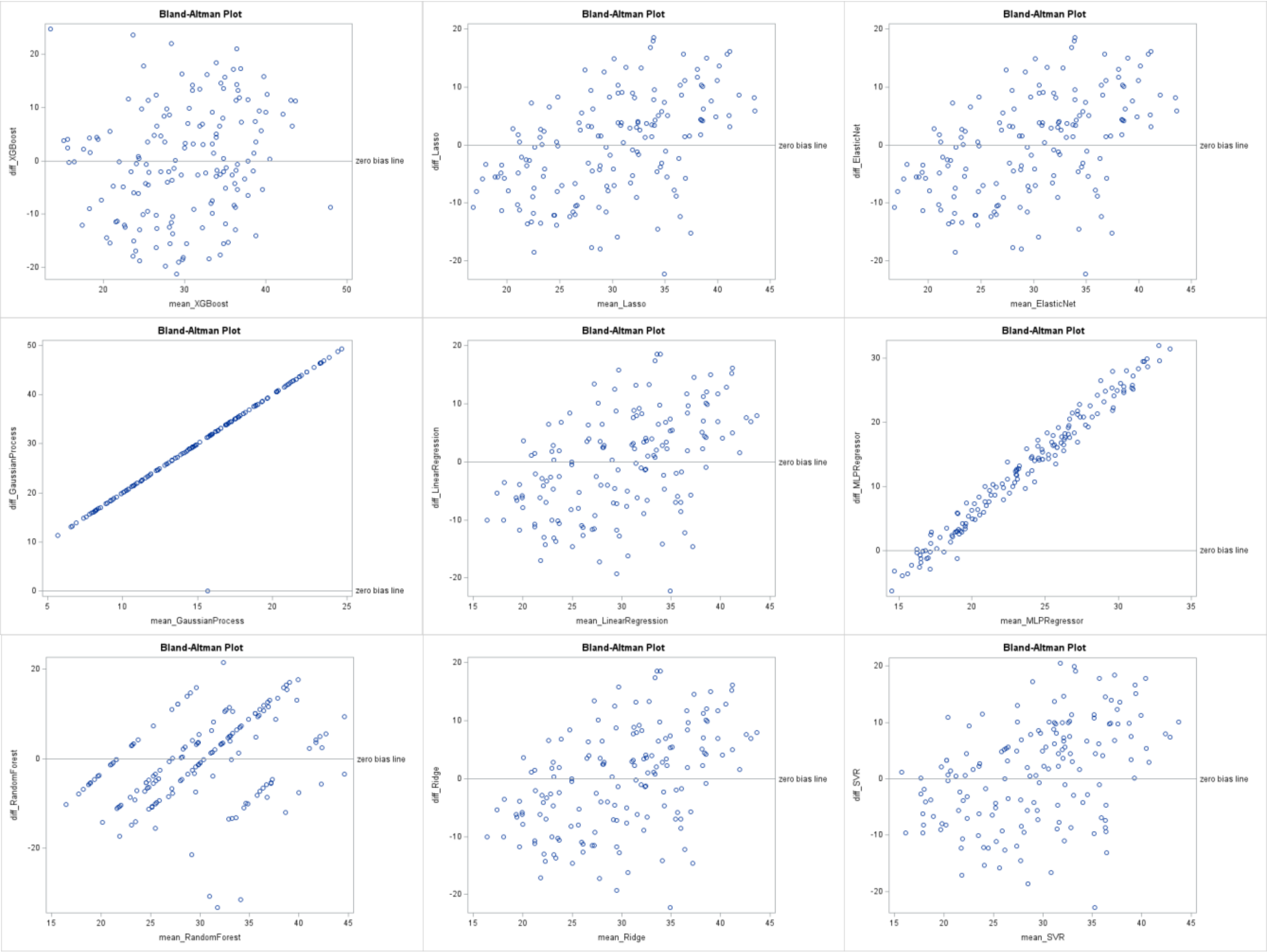

Supplementary Figure S1. Bland-Altman plots showing the agreement between actual and predicted treatment durations using raw data.

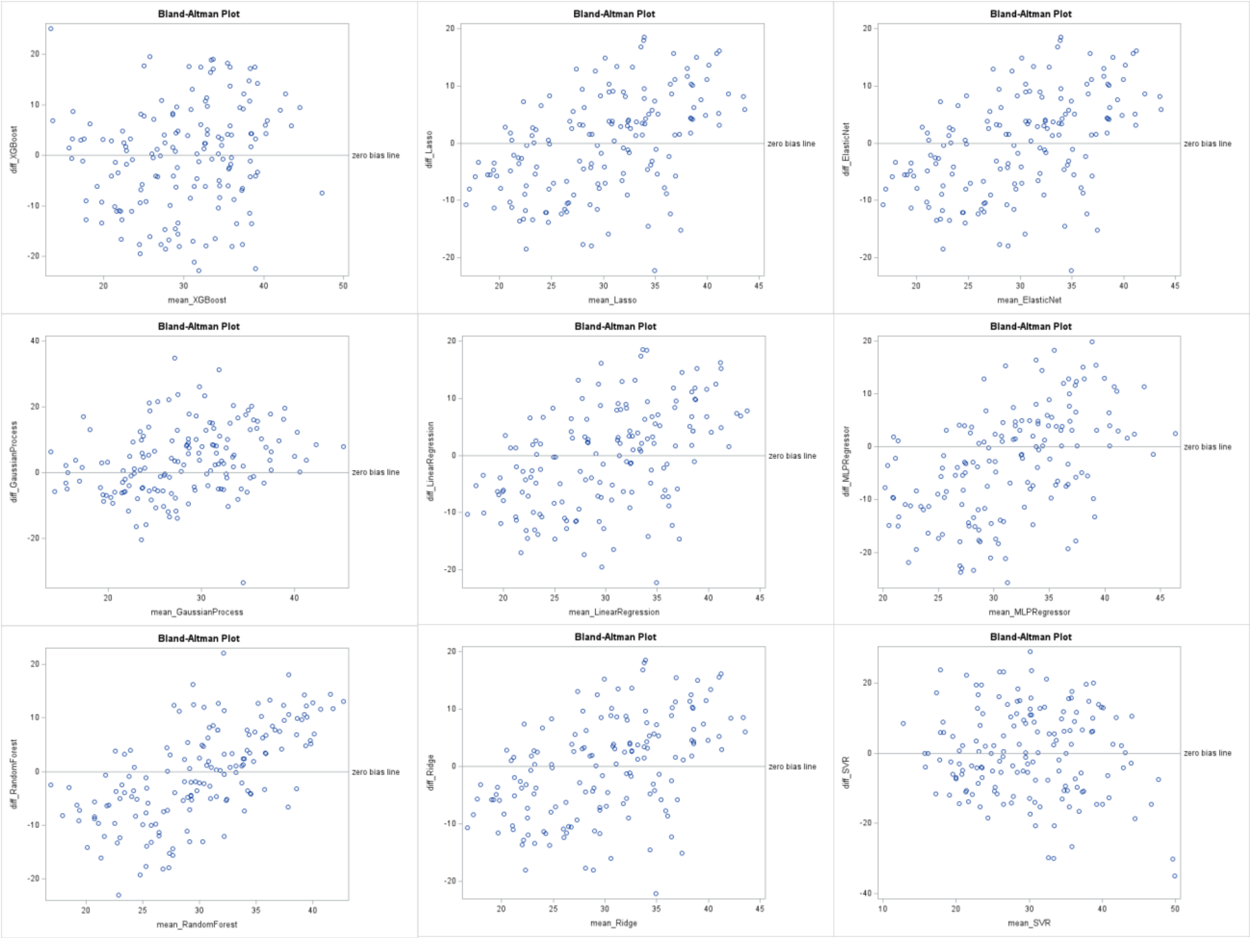

Supplementary Figure S2. Bland-Altman plots showing the agreement between actual and predicted treatment durations using normalized data.
